# 2356. Close correlation of RBD with Live Viral Neutralising Capacity Across Variants and Time Supports Use of RBD Thresholds to Predict Protective Host Immunity

**DOI:** 10.1093/ofid/ofad500.1977

**Published:** 2023-11-27

**Authors:** Grace Kenny, Sophie R O’Reilly, Neil Wrigley Kelly, Riya Negi, Colette Marie Gaillard, Dana Alalwan, Gurvin Saini, Tamara Alrawahneh, Nathan Francois, Matthew Angeliadis, Alejandro García-León, Willard Tinago, Eoin R Feeney, Aoife Cotter, Eoghan de Barra, Obada Yousif, Mary Horgan, Peter Doran, Rebecca J Cox, Donal O’Shea, Ole Olesen, Alan Landay, Andrew Hogan, Jean-Daniel Lelièvre, Virginie Gautier, Oliver A Cornely, Patrick Mallon

**Affiliations:** 1Centre for Experimental Pathogen Host Research (CEPHR), University College Dublin, Belfield, Dublin 4, Ireland2Department of Infectious Diseases, St Vincent’s University Hospital, Elm Park, Dublin 4, Ireland, Dublin, Dublin, Ireland; Centre for Experimental Pathogen Host Research (CEPHR), University College Dublin, Dublin, Dublin, Ireland; Maynooth University, Dublin, Dublin, Ireland; 1Centre for Experimental Pathogen Host Research (CEPHR), University College Dublin, Belfield, Dublin 4, Ireland, Dublin, Dublin, Ireland; 1Centre for Experimental Pathogen Host Research (CEPHR), University College Dublin, Belfield, Dublin 4, Ireland, Dublin, Dublin, Ireland; Centre for Experimental Pathogen Host Research (CEPHR), University College Dublin, Dublin, Dublin, Ireland; 1Centre for Experimental Pathogen Host Research (CEPHR), University College Dublin, Belfield, Dublin 4, Ireland, Dublin, Dublin, Ireland; Centre for Experimental Pathogen Host Research (CEPHR), University College Dublin, Belfield, Dublin 4, Ireland, Dublin, Dublin, Ireland; Centre for Experimental Pathogen Host Research (CEPHR), University College Dublin, Belfield, Dublin 4, Ireland, Dublin, Dublin, Ireland; Centre for Experimental Pathogen Host Research (CEPHR), University College Dublin, Belfield, Dublin 4, Ireland, Dublin, Dublin, Ireland; Centre for Experimental Pathogen Host Research (CEPHR), University College Dublin, Belfield, Dublin 4, Ireland, Dublin, Dublin, Ireland; Centre for Experimental Pathogen Host Research, University College Dublin, Belfield, Dublin, Ireland; Centre for Experimental Pathogen Host Research (CEPHR), University College Dublin, Dublin, Dublin, Ireland; 1Centre for Experimental Pathogen Host Research (CEPHR), University College Dublin, Belfield, Dublin 4, Ireland5Department of Infectious Diseases, Mater Misericordiae University Hospital, Eccles St, Dublin 7, Ireland, Dublin, Dublin, Ireland; 6Department of Infectious Diseases, Beaumont Hospital, Beaumont, Dublin 9, Ireland7Department of International Health and Tropical Medicine, Royal College of Surgeons in Ireland, Dublin, Ireland, Dublin, Dublin, Ireland; 4Endocrinology Department, Wexford General Hospital, Carricklawn, Wexford, Ireland, Wexford, Wexford, Ireland; 8Department of Infectious Diseases, Cork University Hospital, Wilton, Co Cork, Ireland, Cork, Cork, Ireland; 3School of Medicine, University College Dublin, Belfield, Dublin 4, Ireland, Dublin, Dublin, Ireland; Department of Clinical Science, University of Bergen, Norway, Bergen, Hordaland, Norway; University College Dublin, Dublin, Dublin, Ireland; European Vaccine Initiative, Heidelberg, Germany, Heidelberg, Baden-Wurttemberg, Germany; 9Department of Internal Medicine, Rush University, Chicago, Il, USA, Chicago, Illinois; Kathleen Lonsdale Institute for Human Health Research, Maynooth University, Maynooth, Kildare, Ireland; CHU Henri Mondor, Creteil, Ile-de-France, France; 1Centre for Experimental Pathogen Host Research (CEPHR), University College Dublin, Belfield, Dublin 4, Ireland, Dublin, Dublin, Ireland; University of Cologne, Cologne, Germany, Cologne, Nordrhein-Westfalen, Germany; University College Dublin, Dublin, Dublin, Ireland

## Abstract

**Background:**

Although SARS-CoV-2 neutralising antibodies protect against severe COVID-19, whether circulating IgG titres reflect underlying host capacity to neutralise wild type (WT) and variants of concern (VOC) and what IgG threshold reflects sufficient neutralising capacity remains unclear.

**Methods:**

In plasma from individuals in the All Ireland Infectious Diseases Cohort, we measured anti receptor binding domain (RBD) IgG and neutralising capacity by a micro-neutralisation assay, which determined the maximum plasma dilution to maintain 50% inhibition of replication (NT50) of live SARS-CoV-2, using WT and VOC (Beta and Omicron). We examined 3 groups sampled at 3 time periods: unvaccinated, prior infected (group 1, WT dominated), primary (2 dose) vaccinated (group 2) and group 3 with hybrid immunity (booster vaccine and Omicron infection). Although an NT50 ≥100 IU is considered sufficient protection in vaccine trials, as some VOC induce up to 6-fold reduction in NT50, we used NT50 ≥ 1000 IU as a cut off for WT neutralisation that would retain neutralisation against VOC. We used ROC curves to explore sensitivity and specificity and Youden Index to determine the RBD threshold associated with WT NT50 < 1000 IU. We validated the accuracy of this RBD threshold to predict NT50 < 100 IU against Beta and Omicron in groups 2 and 3 respectively.

**Results:**

We included 255 participants across the 3 groups (table 1). In all groups, RBD highly correlated with WT NT50 (rho 0.81, 0.76 and 0.77 in groups 1, 2 and 3, all p< 0.001). In group 1 Youden’s Index determined RBD < 456 BAU/ml to best predict WT NT50 < 1000 IU (sensitivity 77% (95% CI 69, 84%), specificity 100% (95% CI 82, 100%)). All 99 samples with RBD < 456 BAU/ml had WT NT50 < 1000 IU; positive predictive value (PPV) 100%, (95% CI 95, 100%), and overall accuracy 80% (95% CI 73, 86%).

When validated in groups 2 and 3, RBD < 456 BAU/ml retained good accuracy at predicting underlying neutralisation against VOC (group 2, accuracy 80% (95% CI 67, 90%) in predicting NT50 < 100 IU against Beta, and in group 3, overall accuracy of 87% (95% CI 77, 94%) to determine an NT50 of < 100 IU against Omicron.

**Table 1:**
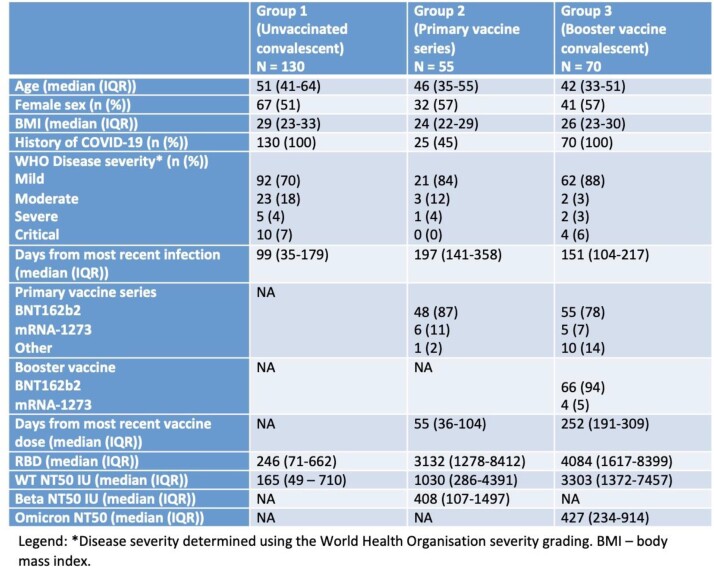
Participant Characteristics

**Figure:**
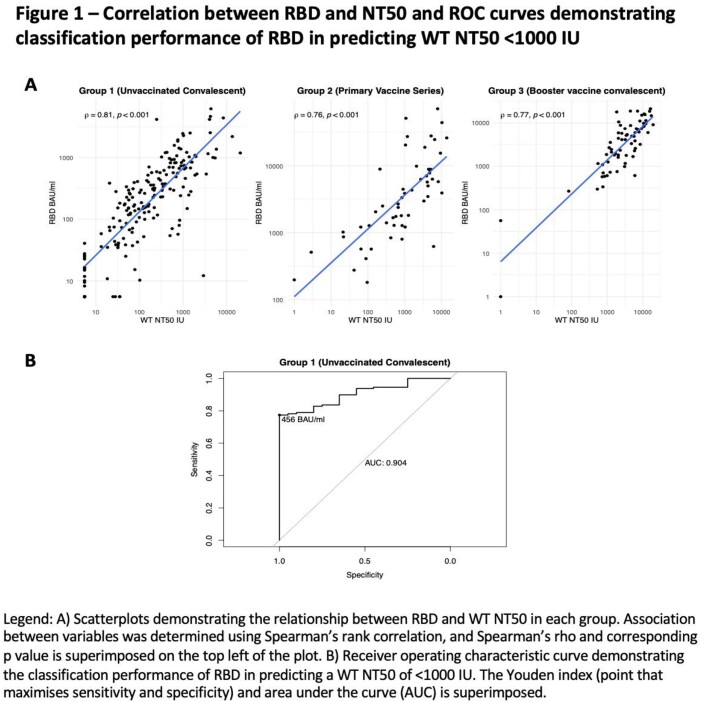

**Conclusion:**

Host RBD titres closely correlate with NT50. An RBD threshold of < 456 BAU/ml accurately predicts a clinically relevant neutralising capacity against both WT and common VOC.

**Disclosures:**

**Oliver A. Cornely, MD PhD**, DZIF: Advisor/Consultant|DZIF: Board Member|DZIF: Grant/Research Support|DZIF: Honoraria|DZIF: Stocks/Bonds

